# Epithelioid Sarcoma: Opportunities for Biology-Driven Targeted Therapy

**DOI:** 10.3389/fonc.2015.00186

**Published:** 2015-08-17

**Authors:** Jonathan Noujaim, Khin Thway, Zia Bajwa, Ayeza Bajwa, Robert G. Maki, Robin L. Jones, Charles Keller

**Affiliations:** ^1^Royal Marsden Hospital, London, UK; ^2^Children’s Cancer Therapy Development Institute, Fort Collins, CO, USA; ^3^Adult and Paediatric Sarcoma Program, Tisch Cancer Institute, Mount Sinai School of Medicine, New York, NY, USA

**Keywords:** epithelioid sarcoma, SMARCB1, INI1, BAF47, SWI/SNF complex

## Abstract

Epithelioid sarcoma (ES) is a soft tissue sarcoma of children and young adults for which the preferred treatment for localized disease is wide surgical resection. Medical management is to a great extent undefined, and therefore for patients with regional and distal metastases, the development of targeted therapies is greatly desired. In this review, we will summarize clinically relevant biomarkers (e.g., *SMARCB1*, CA125, dysadherin, and others) with respect to targeted therapeutic opportunities. We will also examine the role of EGFR, mTOR, and polykinase inhibitors (e.g., sunitinib) in the management of local and disseminated disease. Toward building a consortium of pharmaceutical, academic, and non-profit collaborators, we will discuss the state of resources for investigating ES with respect to cell line resources, tissue banks, and registries so that a roadmap can be developed toward effective biology-driven therapies.

## Introduction

**Figure 1:**
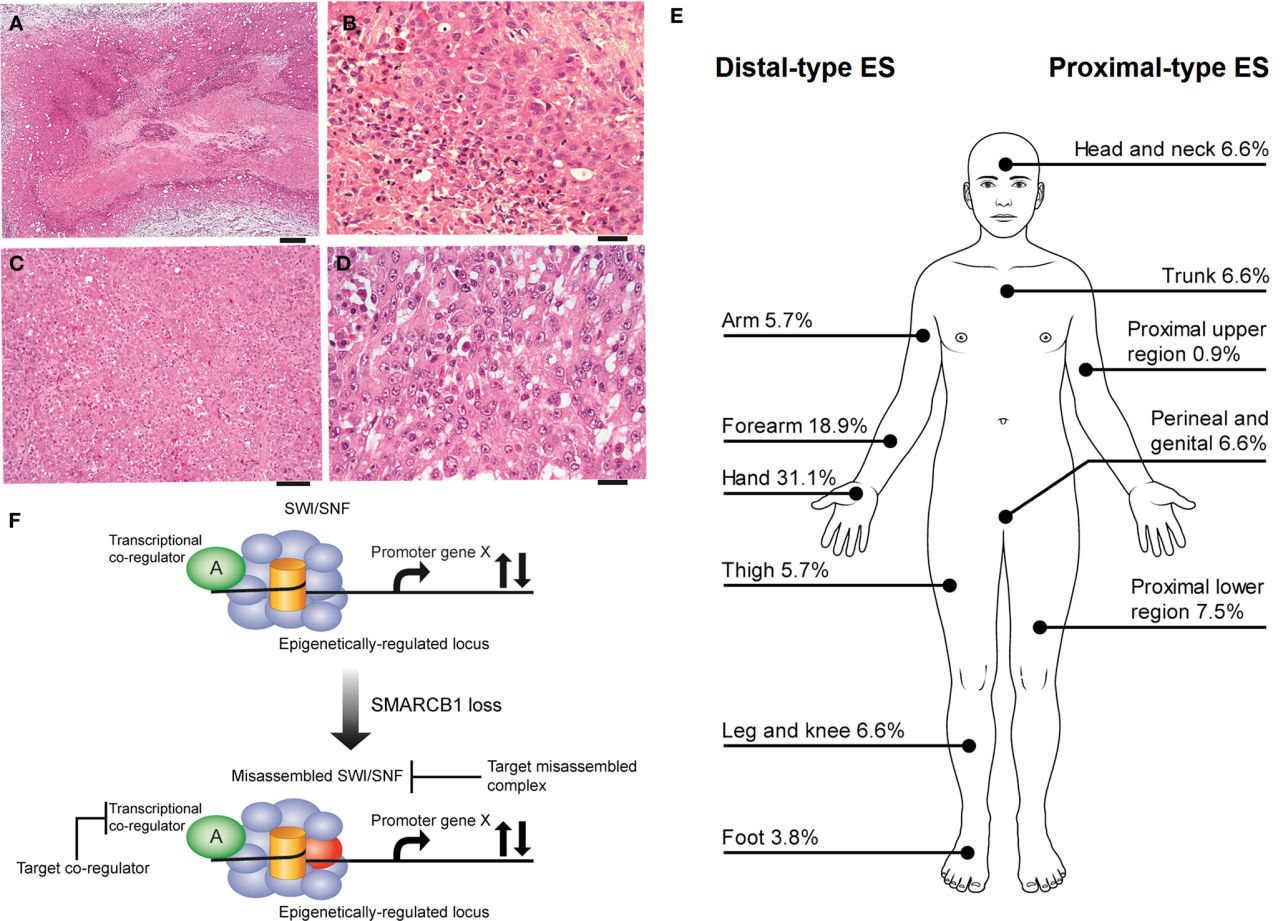
**(A,B)** Distal-type ES. **(A)** Low power histology shows a nodule of tumor present in the dermis and subcutis, comprising a large area of central geographic necrosis, surrounded by sheets of relatively uniform polygonal neoplastic cells (hematoxylin and eosin, ×40). Scale bar, 500 μM. **(B)** At higher power, these are medium-sized, rounded cells, with ovoid vesicular nuclei with even chromatin, and small nucleoli. This example is cellular, but more sparsely cellular neoplasms can appear subtle, and the neoplastic cells may be confused with inflammatory cells. The characteristic necrosis is seen abutting the tumor cells (bottom left of field) (hematoxylin and eosin, ×200). Scale bar, 50 μM. **(C,D)** Proximal-type ES. **(C)** At low power, proximal-type ES comprises sheets or lobules of medium-sized to large round cells, and is seen to lack the more defined architecture and geographic central necrosis of the distal-type variant (hematoxylin and eosin, ×40). Scale bar, 20 μM. **(D)** At higher power, this is characterized by a sheet-like growth of large polygonal cells, often with focal rhabdoid morphology, and which have ovoid vesicular nuclei, prominent large nucleoli, and relatively abundant eosinophilic cytoplasm. The cells are often more pleomorphic than those of the distal-type variant. On morphology alone, these cells are difficult to distinguish from other malignant epithelioid cells, such as those of carcinoma, melanoma, rhabdomyosarcoma, or epithelioid angiosarcoma, and therefore immunohistochemistry is crucial for establishing a correct diagnosis (hematoxylin and eosin, ×200). Scale bar, 50 μM. **(E)** Distributions of ES subytpes, adapted from the largest series reported by the French Sarcoma Group (9). **(F)** Vulnerabilities in the misassembled SWI\SNF complex when *SMARCB1* is absent. Using epithelioid sarcoma as well as rhabdoid tumor as a basis for this model of *SMARCB1* null tumors, the misassembled SWI/SNF complex has the potential to dysregulate target loci that may be co-regulated by other transcription factors (36, 38–40, 43) and thereby present indirect ways to drug target the misassembled complex.

Epithelioid sarcoma (ES), first described by Enzinger over half a century ago (1), is a rare neoplasm accounting for <1% of adult soft tissue sarcomas and between 4 and 8% of pediatric non-rhabdomyosarcomatous sarcomas (2, 3). ES is presumed to be a mesenchymal malignancy, but ES characteristically exhibits both mesenchymal and epithelial markers. The cell of origin and molecular drivers are still a matter of debate. ES is divided into two recognizable clinicopathological entities, classic ES (also called distal-type ES), and proximal-type ES (Figures 1A–E). These two subtypes are thought be a continuum of disease rather than distinct entities (4). Distal-type ES is histologically identifiable by tumor nodules with central necrosis surrounded by large polygonal cells and spindle cells merging in the periphery (5) (Figures 1A,B). Described variants include angiomatoid variant, fibroma-like variant, and myxoid variant. Proximal-type ES is characterized by a multinodular pattern and sheet-like growth of large polygonal cells, often accompanied by a focal or predominant rhabdoid morphology (6) (Figures 1C,D). A specific marker has not yet been identified in ES. On immunohistochemistry (IHC), virtually all cases are positive for cytokeratin (CK) and epithelial membrane antigen (EMA) and most cases co-express vimentin. The marker CD34 is expressed in 60–70% of cases. IHC studies are typically negative for S-100, neurofilament protein, carcinoembryonic antigen, factor VIII-related antigen and CD-31, and INI-1 (*SMARCB1*) whose expression is lost in tumor nuclei (7). Establishing a diagnosis of ES can be difficult as tumors can present with a wide range of appearances and immunophenotypes. The differential diagnosis include fibrous histiocytoma, nodular fasciitis, other reactive proliferations, fibromatosis, giant cell tumor of tendon sheath, sclerosing epithelioid fibrosarcoma, and even some carcinomas and melanomas (7). IHC is helpful in differentiating these entities. Epithelioid vascular tumors can resemble ES and efforts must be made to exclude a diagnosis of epithelioid hemangioendothelioma. In epithelioid hemangioendothelioma, the unique translocation t(1;3)(p36;q25), resulting in the fusion of *WWTR1* with *CAMTA1I* establishes a firm diagnosis (8).

The reported overall peak incidence of ES is around 35 years of age (9, 10). Distal-type ES is more frequently diagnosed and tends to affect a younger (20–40 years of age) and more predominantly male population compared to proximal-type ES, which is usually found in an older population (20–65 years of age) (9, 11). Distal-type ES can present itself as superficial, slow growing painless firm nodules leading to chronic non-healing ulcers affecting mostly the hands and arms. Distal-type ES can also arise as deep-seated slow growing tumors in the extremities or in the tenosynovial tissues. Proximal-type ES is more often diagnosed as deep infiltrating soft tissue masses affecting axial proximal regions and is thought to be associated with a more aggressive course (6). Figure 1E illustrates the sites of involvement of disease. In the largest reported cohort, a majority of ES patients (47%) had localized disease at presentation (2). ES is one of the rare sarcomas that regularly spread to lymph nodes (2, 12, 13). The course of disease is characterized by multiple local recurrences and eventual metastatic spread in 30–50% of cases with the lungs being the primary site of involvement (11). It might be said that local recurrence is the gateway to metastasis.

## Management and Prognosis

Optimal management of this rare sarcoma remains to be defined. The cornerstone of treatment of localized disease is wide surgical resection (14). Neo-adjuvant or adjuvant radiation therapy is often administered in an attempt to reduce local relapses (15, 16). The role of adjuvant chemotherapy is unclear (13, 14, 17, 18). Despite multimodal management, the relapse rate remains high and recurrences tend to occur many years later following initial therapy. Reported local relapse rates are ~35% (11, 18, 19). Patients with localized disease fare better compared to regional disease [5-year overall survival (OS) of 75 vs. 49%]. Pediatric patients seem to have a favorable prognosis [5-year OS of 92.4%] as they are more likely to be diagnosed with localized distal-type ES and are less likely to have nodal or metastatic involvement at presentation (3).

Even though reasonable control of localized disease is possible, metastatic spread is seen in approximately half of patients (2). The available literature on palliative chemotherapy in ES is limited to case reports and small retrospectives studies. The most commonly administered chemotherapy regimens are single-agent anthracycline therapy or the combination an anthracycline with ifosfamide (20). A single group reported activity of a regimen combining gemcitabine with docetaxel, but the experience is limited to a small number of patients (21). The activity of navelbine was raised in a case report and may warrant further investigation (22). Partial responses are rare. Most patients achieve stable disease at best with palliative chemotherapy. In one study, tumor regression was only seen in distal-type disease (20). However, another group reported high-response rates in proximal-type ES using doxorubicin-ifosfamide combination (23). With the medical evidence being limited to small retrospectives studies, it is difficult to draw definitive conclusions regarding the chemosensitivity of this histological subtype.

Despite the administration of palliative chemotherapy, patients with metastasis have a poor prognosis. The reported median survival is ~52 weeks and the 1- and 5-year survival rates are 46 and 0%, respectively (2, 20). Therefore, a substantial unmet need exists to improve the medical management of ES patients by establishing novel systemic regimens and exploring novel targeted therapy. In this review, we will summarize our current understanding of the underlying biology of this rare disease by highlighting implicated signaling pathways and potential actionable biomarkers (Table 1). In order to establish a roadmap that can be developed toward effective biology-driven therapies, we will highlight therapeutics opportunities and drugs with promising activity.

**Table 1:** **Potential actionable biomarkers in clinical epithelioid sarcoma samples**.

Biomarker	Clinical relevance and incidence of biomarker	Available/potential diagnostic	Reference
p53	84% moderate-high nuclear expression by IHC	IHC via TMA	(73)
Cyclin D1	96% expression by IHC	IHC, FISH	(89)
	0% amplification by FISH		
EGFR	77%-93% expression by IHC; absence of amplification via FISH; absence of mutation by PCR	IHC, FISH, PCR	(59, 66)
VEGF-A	73% by IHC	IHC via TMA	(73)
VEGF-C	96% by IHC	IHC via TMA	(73)
mTOR (via p4EBP1 and pSRP)	100% expression by IHC via TMA	IHC via TMA	(59)
PTEN	Loss of expression in 40% by IHC via TMA	IHC via TMA	(59)
β-Catenin	31% nuclear expression by IHC; 81% cytoplasmic expression by IHC	IHC via TMA	(73)
Interleukin2-Rβ	86% expression by IHC	IHC	(90)
SMARCB1 (INI1)	Lost expression in 85–93% by IHC; 21% mutation by PCR	IHC, FISH, PCR	(9, 49, 50, 91, 92)
SALL4	Expression in 24% of proximal-type by IHC	IHC	(93)
ERG	Expression in 38–68% by IHC; no found rearrangement by FISH	IHC, FISH	(93–95)
FLI1	95% expression by IHC	IHC	(94)
PBRM1	Lost expression in 83% by IHC	IHC	(96)
GLUT1	Expression in 50% by IHC	IHC	(91)
NRAS	Mutated in one case report by sequence assay	Sequence assay	(97)
Dysadherin	54% expression by IHC	IHC	(70)
E-cadherin	Absent expression	IHC via TMA	(70, 73)
SYT-SSX1	Low expression by RT-PCR in one patient; negative by FISH	RT-PCR	(98)

## Cytogenetic Analyses

Cytogenetic analyses were first attempted to better understand the biology of ES (24–29). The karyotype analysis on clinical samples or cells lines varied greatly and was mostly done in adult cases. A minority of samples were diploid, some polypoid, while a great majority had complex patterns consisting of multiple numerical and structural rearrangements (see Table 3). Pediatric cytogenetic analyses seem to indicate less complex genetic alterations compared to adults and may therefore offer an explanation of their more favorable prognosis (30, 31). Translocations t(8;22)(q22;q11) in distal-type ES and t(10;22) in proximal-type ES were found (24, 32). However, compared to other translocation-driven sarcomas, there is no unique identifiable reoccurring cytogenetic pattern in ES. The only identified recurrent breakpoints have been structural rearrangements involving 18q11 and 22q11. The observation that a substantial number of ES had either rearrangements or deletions of 22q led to the hypothesis that this region may contain a tumor suppressor gene (32, 33). Further studies identified *SMARCB1* as being involved in the tumorigenesis of ES (34).

### *SMARCB1* 

The *SMARCB1* gene, located at 22q11, codes for BAF47, a core subunit of the SWI/SNF ATP-dependent chromatin remodeling complex and acts as a tumor suppressor gene (35). Components of the SWI/SNF complex are mutated in 20% of cancers, most notably rhabdoid tumor (36). This complex regulates genes by enabling the nucleosome to reposition itself in relation to the DNA sequence (37). Inactivation of *SMARCB1* leads to neoplastic transformation by transcriptional deregulation of target genes implicated in regulating genomic stability, cell-cycle progression, and other signaling pathways in cooperation with transcriptional co-regulators (e.g., MyoD, Olig2) (36, 38, 39). *SMARCB1* was shown to transcriptionally regulate p16INK4a and/or p21 and repress cyclin D1, thereby suppressing E2F activity and its target genes (40–42). *SMARCB1*-deficient cells have been implicated to have aberrant Hedgehog signaling pathway activation (40, 43). Brenca et al. demonstrated that loss of *SMARCB1* expression in the ES cell line VAESBJ was caused by homozygous deletion of *SMARCB1* through mutations of exon 1. They also identified equally prevalent homozygous deletion of *CDKN2A* and *CDKN2B* loci, responsible for encoding p16, p14, and p15 proteins. Restoration of *SMARCB1* led to a reduction of cell proliferation and cell migration and to an increase in sensitivity to genotoxic stress, thereby providing evidence to support *SMARCB1* inactivation in the tumorigenesis of ES (44). For rhabdoid tumor, SWI/SNF disruption is sufficient to cause neoplastic transformation (45). However, in the context of ES, loss of *SMARCB1* by itself is not sufficient. Interestingly, knockout of *SMARCB1* in primary fibroblast cells causes rapid growth arrest and p53-mediated programed cell death (46). However, when mutations of *TP53* co-exist, tumor proliferation is dramatically increased (47). Brenca et al. demonstrated that the VAESBJ cell line retains wild-type *TP53*, but hypothesized that the homozygous loss of *CDKN2A* which leads to impaired p16/RB and p14/TP53 responses likely contributes to the genomic instability seen in this cell line (44). Hence, other signaling pathways may contribute to tumor progression in ES as witnessed by the complex genetic landscape reported in cytogenetic studies. Whether the *SMARCB1*-deficient SWI/SNF complex exists in a misassembled state as it does in rhabdoid tumor (48), and to what extent the misassembled complex aberrantly deregulates loci that are not normally associated with the SWI/SNF complex remains to be investigated. Most certainly, the milieu of transcriptional co-regulators in ES will be different than in rhabdoid tumor.

Targeting *SMARCB1* is complicated by the different mechanisms of loss of expression. IHC studies demonstrated that the loss of expression of *SMARCB1* ranges from 85 to 93% of cases (9, 34, 49, 50). Allelic homozygous deletions varied from 5 to 71%; however, the true value may be ~10% (51–53). Papp et al. identified different mechanisms to explain the loss of expression of *SMARCB1*: 13% of cases had biallelic deletions, 33% showed single-allelic deletion, and 4% had point mutations (52). In 59% of cases, both alleles were intact and no cases had promoter hypermethylation nor post-translational modification. The authors went on to show that loss of *SMARCB1* protein expression in those cases is due to epigenetic gene silencing by oncomiRs. Three of the overexpressed miRNAs (miR-206, miR-381, and miR-671-5p) could silence the *SMARCB1* mRNA expression in cell cultures (54). The role of oncomiRs was also validated by another group where miR193a-5p could equally inhibit the mRNA expression of *SMARCB1* (55). Beyond targeting the misassembled SWI/SNF complex, transcriptional co-regulators are also theoretical targets (Figure 1F). In summary, loss of *SMARCB1* has a crucial role in the pathogenesis of ES (along with other signaling pathways) and therefore is an interesting target to pursue for the development of new therapies. Acknowledging that the restoration of *SMARCB1* function is likely the primary therapeutic opportunity in ES, in the paragraphs to follow we discuss other therapeutic opportunities related to consistent alterations in other signaling pathways that may also contribute to the pathogenesis of ES.

## PI3K–AKT–mTOR Signaling Pathway

The phosphatidylinositol 3-kinase/protein kinase-B/mammalian target of rapamycin (PI3K/AKT/mTOR) signaling pathway has been studied extensively and is activated in a myriad of cancers. This signaling pathway’s signaling regulates cell proliferation, differentiation, cellular metabolism, and cytoskeletal reorganization leading to apoptosis and cancer cell survival (56). A previous study done on *SMARCB1*-deficient tumor cells revealed persistent AKT activation (57). This finding led Imura et al. to further investigate the importance of this signaling pathway in ES (58). By studying two *SMARCB1*-deficient cell lines (VAESBJ and Asra-EPS), this group has shown that AKT/mTOR pathway is constitutively hyperactivated. Results demonstrated that silencing mTOR by transfecting cell lines with anti-mTOR-specific ­siRNAs suppressed cell proliferation. However, inhibition of mTOR with everolimus caused tumor growth delay without shrinkage. Blocking the mTOR signaling pathway with everolimus caused an increase in AKT and ERK activity, which was subsequently shown to be dependent of c-MET activation. Blocking c-MET activation had a variable effect on growth inhibition on studied cell lines. This variability could be partially explained by the degree of loss of PTEN, which is thought to contribute to resistance to c-MET inhibitors through sustained AKT activation upon mTOR blockade. Combining agents to block both AKT and c-MET were more effective in inducing tumor arrest compared to using either one alone. The importance of AKT and c-MET/HGF pathways was also highlighted through immunohistochemical analysis of random clinical samples. The variability of AKT activation and loss of PTEN expression in different cell lines were also confirmed by another group and thought to correlate with sensitivity of rapamycin (59). This heterogeneity could highlight the complex genetics of the disease as well as the variable importance of PI3K/AKT/mTOR signaling pathway in the tumorigenesis of ES. *In vitro* sensitivity to mTOR inhibitors is likely an imperfect surrogate for clinical activity. Nonetheless, these preclinical data are interesting and may warrant additional studies before pursuing clinical trials. Resistance to single-agent mTOR inhibitors can not only be a potential issue but can also possibly be overcome by simultaneously targeting other pathways. These findings are consistent with the shortcomings of targeting mTOR signaling pathway in general and highlight the importance of patient selection and identification of putative biomarkers (60).

## EGF Pathway

The human epidermal growth factor signaling pathway regroups four distinct receptor tyrosine kinases, HER1 (ErbB-1, EGFR), HER2 (ErbB-2), HER3 (ErbB-3), and HER4 (ErbB-4) and is implicated in cell proliferation, apoptosis, and angiogenesis (61). The role of EGFR in malignant transformation of carcinomas has been extensively studied. Recently, EGFR expression was revealed to be present in soft tissue and bone sarcomas (62, 63). However, subsets of disease demonstrating tyrosine kinase domain mutations were rare (64, 65). These findings sparked an interest in studying EGFR in ES. Cascio et al. showed that 93% of clinical samples (including distal and proximal-type ES) expressed EGFR by IHC (66). This high level of expression of EGFR was also corroborated by Xie et al. (59). Furthermore, Cascio et al. went on to show an absence of EGFR amplification via FISH studies. Sequencing of the *EFGR* gene tyrosine kinase domain revealed no point mutations, insertions, or deletions. Xie et al. further investigated the role of EGFR pathway in the tumorigenesis of ES. EGF-induction contributes to cell-cycle progression partly through upregulation of cyclin D1. EGFR activation also causes an increase in migration and invasion of ES cells where high levels of expression of MMP2 and MMP9 were found. Next, this group tested whether EGFR inhibition with erlotinib would be a viable therapy. Exposure to erlotinib caused tumor growth delay without causing tumor arrest. An explanation for this incomplete response is given by the cooperation of HGFR/MET pathway with EGFR in sustaining AKT and ERK phosphorylation. Dual inhibition of both those pathways had a synergistic effect in decreasing cell proliferation (44). Combining inhibition of EGFR pathway with erlotinib and mTOR pathway with rapamycin also proved to be synergistic causing cell-cycle arrest as well as an increase in apoptosis (59). Targeting solely the EGFR pathway may not translate to a possible clinical benefit. However, combined inhibition of EFGR with either mTOR or HGFR\MET may worth investigating further through preclinical animal studies.

## Other Possible Actionable Pathways and Targets

Dysadherin is a cancer-associated cell membrane glycoprotein shown to downregulate E-cadherin cell-mediated adhesion and to promote metastasis (67). Dysadherin contributes to metastatic progression through autocrine activation of CCL2 expression in part through activation of the nuclear factor-kappaB pathway (68). Dysadherin also has the ability to attribute stem-cell like properties to cancer cells (68, 69). Higher mRNA expression levels of dysadherin were documented in cell lines derived from proximal-type ES compared to distal-type ES (70). This difference in expression in levels of dysadherin may offer a possible explanation to the poor prognosis associated with proximal-type ES. Interestingly, in breast cancer cell lines, dysadherin overexpression was shown to possibly enhance AKT activation. Subsequently, inhibiting AKT reduced dysadherin’s ability to promote cell mobility and tumor cell invasion (71). Targeting dysadherin could be potentially exploited to treat ES, but further work is needed. Agonists of the CCL2 receptor, CCR2, such as PF-04634817, may be one area to begin investigation.

The role of Wnt/β-catenin signaling pathway in cancer is well documented. APC deficiency or β*-catenin* mutations preventing its degradation lead to constitutive activation of β-catenin signaling, which in turn contribute to stem-cell renewal and proliferation (72). In ES, IHC studies revealed low expressions of nuclear β-catenin (73). Furthermore, no β*-catenin* gene mutations were found (74). Therefore, the proliferative abilities of ES cells are probably related to other mechanisms than Wnt/β-catenin signaling pathway. This finding is in contradistinction to the β-catenin activation seen in rhabdoid tumor (39). Interestingly, IHC studies identified a complete loss of E-cadherin (70, 73). E-cadherin is a calcium-dependent glycoprotein responsible for cell–cell adhesion (75). E-cadherin/β-catenin protein complexes have an active role in epithelial-to-mesenchymal transition (EMT), an important mechanism for the subsequent development of metastasis (76, 77). Further studies are needed to elucidate the importance of loss of adhesion molecules in tumor progression in ES.

CA125 was first identified and used as a serum marker for epithelial ovarian carcinoma (78). IHC studies revealed high positivity and specificity of CA125 in ES compared to other sarcomas (79). High expressions of the *MUC16* gene were identified by RT-PCR in ES cell lines. Serum levels of CA125 also seem to correlate with disease progression (80). Measuring CA125 serum levels is well-established and routinely available and could potentially be useful in monitoring disease status and evaluating response to therapy. Targeted immune strategies toward CA125 and MUC16 are active areas of research in ovarian cancer and any potential breakthroughs could possibly be applicable in treating ES (81, 82).

## Tyrosine Kinase Inhibitors

As stated previously, chemotherapy has a limited role in the management of ES. Early studies explored the reasons underlying chemotherapy resistance. A study of the SARCCR2 cell line showed overexpression of P-glycoprotein, an ATP-binding cassette (ABC) chemotherapy export pump. Using verapamil and cyclosporine A to reverse multidrug resistance, the authors showed increased sensitivity to doxorubicin and vincristine (83). For the GRU cell line, expression of P-glycoprotein and MRP could also be observed (84). However, one study identified an absence of expression of P-glycoprotein and MRP in the ES-OMC-MN and SFT-8606 cell lines (85). In contradistinction, these studies demonstrated the presence of lung resistant protein (LRP), which mediates multidrug resistance (MDR). Results showed that reversing MDR with cyclosporin A increased sensitivity to actinomycin D, vincristine, and adriamycin. The use of tyrosine kinase inhibitors, the newest ABC inhibitors, to reverse multidrug resistance remains unexplored and may potentially enhance the efficacy of chemotherapy in ES.

The medical evidence for the utility of tyrosine kinase inhibitors impacting ES is scarce. To our knowledge, only one case was reported in the medical literature. Sunitinib showed reasonable disease stabilization in a patient with metastatic ES (86). The underlying reasons why sunitinib was active in this patient are unknown and cannot be explained with what is currently known about the biology of this disease. Pazopanib, a recent oral tyrosine kinase inhibitor approved for the treatment of soft tissue sarcoma (87), is worth investigating prospectively as its activity is similar to sunitinib. Work is still needed in mapping out active signaling pathways and identifying actionable tyrosine kinase domain mutations. Polykinase inhibitors remain therefore greatly unexplored in the management of ES and may one day improve outcome.

## Future Perspectives

Researching and developing new treatment strategies in rare cancers is a challenge, but possible with technology and resources available today and regulatory agency incentives (88). ES is a perfect model to envision what personalized medicine promises for the future. The intent of this review was to draw a roadmap to develop efficient biology-driven therapy. Achieving this will start with the selection of representative cell lines and mouse models of ES (Available cell lines and potential actionable targets are summarized in Tables 2 and 3). Many of the potential targets highlighted in this article were based on IHC-expression or reverse-transcriptase PCR studies. DNA deep-sequencing projects may demonstrate underlying genomic amplification and mutations that can be targeted. Partnership with pharmaceutical companies would allow screening of thousands of compounds on selected cell lines presenting mutations or other actionable targets. Active drugs may then undergo preclinical testing. Those most promising can be prioritized for clinical trials. Drugs being developed in other cancers that share common signaling pathways aberrations with ES may also prove to be useful. It is possible to perform basket trials in rare cancers, and this could be a way of evaluating novel agents in this extremely rare disease. On the way of developing new therapies, possible pitfalls can be expected. As demonstrated on ES cell line models, targeting a single signaling pathway may be insufficient. The complexity of the genetic landscape and the crosstalk between multiple signaling pathways contribute to resistance. This can be overcome by targeting multiple signaling pathways simultaneously. Only through international collaboration between pediatric and adult units, we can remain hopeful that targeted and immune therapy will have a major impact in the management of ES in the near future.

**Table 2:** **Epithelioid sarcoma potential targets and corresponding experimental model systems**.

Biomarker	Human cell line(s)	Reference
ALK	YCUS-5	(31)
AKT	VAESBJ, Epi544	(58, 59)
c-MET	ASRA-EPS, VAESBJ	(58)
Dysadherin	HS-ES-1M, YCUS-5, ES-OMC-MN, SFT-8606	(70)
EGFR	VAESBJ, Epi544, GRU-1	(59, 99)
HGFR/MET	VAESBJ	(44)
IL-6 and IL-6R	ES-OMC-MN	(100)
LRP	ES-OMC-MN, SFT-8606	(85)
Metal free protoporphyrin IX	Va-es-bj	(101)
MMP-2, MMP-9, TIMP-1, TIMP-2, TIMP-4	GRU-1	(59, 102)
MUC gene	FU-EPS-1, SFT-8606	(80)
mTOR	VAESBJ, Epi544	(59)
PDGF	GRU-1	(99)
RAR-α, RAR-β, and RAR-γ	GRU-1	(103)
TGF-α	GRU-1	(99)
TGF-β/Smad signaling and CD 109	ESX	(99, 104)
TNF receptors	GRU-1	(103)
Tyrosine hydroxylase gene (TH)	YCUS-5	(31)

**Table 3:** **Demographic and biological features of human epithelioid sarcoma cell lines**.

Cell line name	Histology	Age (years)	Sex	Primary site	Metastatic	Cell line source	Select chromosomal marker(s)	Mutation(s)	Primary reference (PMID or other)	Related references (PMID or other)	Originating investigator (and institution) or commercial source(s)	Reference
RM-HS1		37	M	Left foot					2432306		Reeves	(105)

HX 165 c		28	M	Penile		Local recurrence			3179184		Kelland (Institute of Cancer Research, UK)	(106)

GRU-I		32	F	Left buttock	Yes	Para-iliac lymph-node			1688830	7525493	Gerharz (University of Mainz)	(107)

SARCCR 2		33	F	Knee		Local recurrence	Chromosomes 13, 14, 16, 18, and 22 were deleted in all cells		8099901		Roché (Centre Claudius Regaud)	(83)

HS-ES-1M	Proximal-type	60	M	Right perineum nodule	Yes	Local recurrence	All exhibited the identical abnormal karyotype of 46, XY, 1i(8)(q10),221, del(22)(q12)		9216728		Sonobe (Kochi Medical School)	(28)

ES020488		26	M		Yes	Cutaneous metastasis	39–83 chromosomes, with various abnormalities but no specific pattern		7685133		Sonobe (Kochi Medical School, Japan)	(108)

Va-es-bj		41	M	Epidural tumor	Yes	Bone marrow aspirate	Chromosomal triploidy with marker chromosomes		21552805	8572585	Helson (St Agnes Hospital)	(109)

ES-OMC-MN	Distal-type	44	F	Right leg nodule	Yes	Chest wall	Modal chromosome number was 45, X, in 74% of metaphases. Other chromosome numbers were 47, XXX, in 14% of metaphases, and 46, XX, in 12% of metaphases		9143739		Kusakabe (Osaka Medical College)	(100)
							Except for a number of sex chromosomes, the chromosomes had no chromosomal anomaly					

YCUS-5	Proximal-type	3	F	Neck mass	no	Neck mass	48, XX, t(2;7)(p23;q32 ~ 34), ?del(6)(q2?5), +7, +8	expression tyrosine hydroxylase gene (TH) expression of ALK	10398195		Goto (Yokohama City University School of Medicine)	(31)

SFT-8606	Distal-type	75	M	Left elbow	yes	Primary tumor	Complex numerical and structural aberrations, including add(8)(p23), add(9)(p13), der(12)t(12;14)(p13;q22), +i(21)(q10), der(22)t(18;22)(q11;p11.2)		8908166		Iwasaki (Fukuoka University School of Medicine)	(26)

Stenman cell line		64	M	Left forearm	yes	Axillary lymph node	No <14 different marker chromosomes were found, of which all but four resulted from terminal deletions	Elevated p21 expression was probably due to an overexpression of the N-ras gene	2196989		Stenman (Gothenburg University)	(110)
							Most frequent del(1) (p21-22), found in about 25% of the cells karyotyped					

FU-EPS-1		21	M	Right upper arm	Yes	Axillary node	Hyperdiploid karyotype with the following chromosomal abnormalities: +i(5)(p10), −8, +13, der(13)t(8;13)(q?;p11), +der(19)t(9;19)(?;?), and del(22)(q13). Gains of 5p, 9q, 19q, and 22q and a loss of 8p		16010416		Nishio (Fukuoka University Faculty of medicine)	(111)

NEPS	Classical	32	M	Forearm		Primary tumor			19756736		Hoshino (Niigata University Graduate School of Medical and Dental Sciences)	(80)

Epi-544				Foot			Modal chromosomal number of 45 (range, 42–45), monosomy of chromosomes 2, 8, 13, and X, trisomy of chromosome 5, and the following structural abnormalities: del 7q, del 9q, del 12p, 16q, t(9q;14q), and t(2q;?)		21357725		Sakharpe (University of Texas MD Anderson Cancer Center)	(73)

ESX	Proximal-type	73	F	Left thigh	Yes	Primary tumor	65 ~ 68, X, −X, or -Y, add (X)(q22), +1, add(1) (p32), add(1) (q21), add(1)(q42), add(1)(q42), der(4;10)(q10;q10), add(8)(p11.2), −9, add(9)(p22), der(11)t(11;14)(p13;q13), −13, add(13)(q22), −14, −15, add(16)(p13.1), −17, −18, add(18)(q21), +21, add(22)(q13), +4 ~ 6mar	CD109 mRNA expression	24376795		Emori (Sapporo Medical University School of Medicine)	(104)

Asra-EPS	Angiomatoid ES	67	M	Right elbow mass	No	Primary tumor	Karyotype showed near-tetraploidy with some chromosomal translocations and fragments		23915498		Imura (Osaka University Graduate School of Medicine)	(112)
							No recurrent chromosomal translocation was detected. 90, XXYY, −4, +5, +8, +9, −10, −13, t(13;15), +14, −15, −15, −20, −22, −22, +1mar					

## Conflict of Interest Statement

The authors declare that the research was conducted in the absence of any commercial or financial relationships that could be construed as a potential conflict of interest.
